# Performance of Luminescent Solar Concentrators Integrated with Negative Replica Layers of Leaf Surface Microstructures

**DOI:** 10.3390/ma15072353

**Published:** 2022-03-22

**Authors:** Bing-Mau Chen, Han-Yi Fu, Shang-Ping Ying, Ting-Wei Hsu

**Affiliations:** 1 Department of Electro-Optical Engineering, Minghsin University of Science & Technology, Hsinchu 30401, Taiwan; hank012341@gmail.com; 2 Department of Biological Sciences, National Sun Yat-Sen University, Kaohsiung 804, Taiwan; hanyifu@mail.nsysu.edu.tw

**Keywords:** luminescent solar concentrator, leaf surface microstructure, negative replica layer

## Abstract

In this study, a negative replica layer of leaf surface microstructures was used to cover the top surfaces of semitransparent thin-film luminescent solar concentrators (LSCs) to enhance the concentrators’ performance. With low reflection on the air–glass interface of the glass plate in a thin-film LSC, a negative replica layer enables the scattering of incident sunlight and increases the path of light transmitted into the LSC and the thin phosphor layer at the bottom surface of the LSC. The incident sunlight is therefore more likely to interact with the phosphor particles in the thin-film phosphor layer, thereby enhancing the performance of the LSC. In this study, semitransparent thin-film LSCs with different inorganic phosphors were examined. The experimental results revealed that the optical collection efficiency of semitransparent thin-film LSCs covered with negative replica layers of leaf surface microstructures was higher than that of the semitransparent thin-film LSCs without negative replica layers. Furthermore, the LSCs with negative replica layers with high haze ratios exhibited high optical collection efficiency. Integrating negative replica layers of leaf surface microstructures as semitransparent layers in thin-film LSCs may optimize the application of LSCs in building-integrated photovoltaics (BIPVs).

## 1. Introduction

Harvesting sunlight using photovoltaic (PV) technologies is one of the most promising solutions to the world’s energy demand problem. However, the implementation of such technologies has been impeded by competition between solar power generation and agricultural productivity due to the high number of new installations in the past few years. Building-integrated photovoltaics (BIPVs) are PV materials or systems that are integrated into building envelopes such as roofs, facades, and windows [1,2,3]. Because BIPVs serve as both building components and means of generating electricity, they may be a possible solution to land-use conflicts.

Luminescent solar concentrators (LSCs) consist of semitransparent plates containing or coated with luminescent materials, such as dyes or quantum dots, that have been designed for facile installation within other structures for BIPV applications. The luminescent material absorbs the incoming sunlight and re-emits it in longer wavelengths. A fraction of the re-emitted light undergoes total internal reflection and eventually reaches the edge of the semitransparent plate; this light is collected by the solar cells along the edges [4]. LSCs are intended to replace large PV modules with polymer- or glass-based plate collectors, which are lightweight and adaptable with regard to color, shape, and size. Without the large PV modules, the use of LSCs can also improve the reduction in energy conversion efficiency with increasing temperature throughout the temperature range with extreme limits [5]. In addition, LSCs can concentrate both direct and diffuse light and are therefore capable of operating under cloudy weather conditions or in shady parts of buildings, where conventional PV modules cannot work. Compared with LSCs with typical bulk-plate configurations, thin-film LSCs exhibit some technological advantages due to the possibility of depositing the thin luminescent film as a conventional coating on any semitransparent substrate by means of typical deposition techniques [6,7]. Thus, LSCs are promising as semitransparent components of BIPVs.

White light-emitting inorganic phosphors may be appropriate luminescent materials for LSCs used in BIPV systems. Because of their long lifetimes, photostability at high temperatures, excellent ultraviolet (UV) stability and chemical compatibility, low cost, and simplicity of use, inorganic phosphors are suitable for outdoor usage [8]. However, to encourage the widespread diffusion of thin-film LSCs in BIPV systems, efforts must be made to increase the performance of LSCs utilizing inorganic phosphors [9,10,11,12,13]. In commercial Si-PV modules, the use of surfaces with microlenses, texture or periodical structures on glass shields or encapsulation layers has optimized the harvest of incident light, thereby enhancing the optical efficiency of the modules [14,15,16,17,18]. Similar approaches have also been applied to increase the efficiency of LSCs. Installing an optical microlens on the front surface of the semitransparent plate of an LSC can increase the intensity of incident light to enhance light collection [19]. Nevertheless, identifying appropriate textures or periodic structures remains a challenge in the development of PV technologies. Through evolution, green plants have developed various nanoscopic, microscopic, and macroscopic structures on the surfaces of their leaves; such structures have inspired various scientific innovations. In general, to replicate structures from biological surfaces, a double-transfer fabrication process is used [20,21,22]. The process first involves the fabrication of a negative mold of a plant leaf. After being cured and released from the leaf, the flexible negative mold is used to transfer the structures onto the positive replica. The use of polydimethylsiloxane (PDMS) for casting microstructure templates in double-transfer fabrication processes is key to biomimetic approaches. PDMS is the most widely adopted material in fabricating microstructures and flexible films with negative microstructure because of its high optical transparency and biocompatibility. Therefore, negative microstructures replicated directly from plant leaves by using PDMS could be used to create a suitable texture or periodic structure for PV technologies.

**Figure 1:**
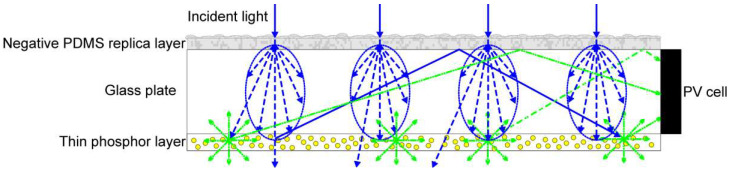
Schematic of a semitransparent thin-film luminescent solar concentrator (LSC) with a negative replica layer and a thin inorganic phosphor layer on the top and bottom surfaces of the glass plate, respectively.

In this study, the effect of negative replica layers of leaf surface microstructures on the performance of semitransparent thin-film LSCs containing commercial phosphors was evaluated. PDMS was used to produce the thin layer with negative microstructures replicated directly from fresh plant leaves. To enhance the harvest of incident light, a thin layer with negative microstructures was fixed on the top surface of a semitransparent thin-film LSC, and the thin phosphor layer was then positioned on the bottom surface of the LSC, as illustrated in Figure 1. The transmittance and optical haze ratio of the negative replica layer of leaf surface microstructures were examined. The performance of the thin layers of the negative replicas of the various leaf surface microstructures on the semitransparent thin-film LSCs was investigated to determine which microstructures yielded the greatest optical collection efficiency.

## 2. Materials and Methods

**Figure 2:**
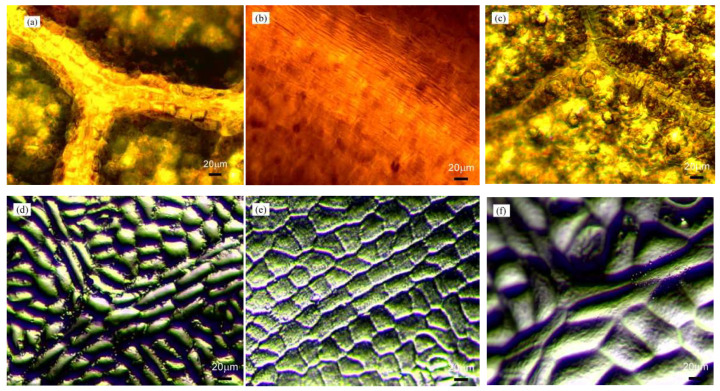
Optical microscope images of the surface morphologies of leaves from (**a**) *Bauhinia*, (**b**) *Epipremnum aureum*, and (**c**) *Pachira macrocarpa* and (**d**–**f**) their respective negative polydimethylsiloxane (PDMS) replica layers.

For the use of negative replica layers with leaf surface microstructures on semitransparent thin-film LSCs, the negative replica layer with a large size was certainly preferred for BIPV applications. Thus, the common large-leafed plants in north Taiwan were chosen in this study. At the meantime, the size and morphology of the surface microstructure were also important for the negative replica layers on semitransparent thin-film LSCs. Then three different large-leafed plants were selected for this study: *Bauhinia* (orchid tree), *Epipremnum aureum* (centipede tongavine) and *Pachira macrocarpa* (lychee). These large leaves were selected according to their sizes (20–70 μm) and the distinct morphologies of their surface microstructures. To maintain their natural surface features and to avoid dehydration artefacts, the leaves were freshly picked just before each replication process [20,21,22]. The fresh leaves were cleaned with ethanol and dried with pressurized air to remove moisture. Thereafter, a negative PDMS replica layer was fabricated directly from each leaf. The chosen plant leaf was cut into small pieces of approximately 5 cm × 5 cm, and a piece was fixed to a clean Petri dish using a double-sided adhesive. Two-component PDMS gel (Sylgard 184, Dow Corning, Midland, MI, USA) with a weight ratio of 10:1 (elastomer to crosslinker) was uniformly mixed and placed in a vacuum chamber for 30 min to remove any air bubbles trapped in the mixture. The mixture was slowly poured onto the chosen piece of leaf, and the PDMS mixture coated the Petri dish. The Petri dish filled with the PDMS was kept in a vacuum chamber for 1 h to remove the air bubbles trapped at the interface between the PDMS and the leaf. The sample was heated in an oven at 60 °C for 2 h to cure the thin PDMS layer, and the negative PDMS replica layer was peeled off the piece of leaf. At least three negative PDMS replica layers were fabricated using each plant to ensure methodical repeatability. To ensure that the optical characteristics for different plants were comparable, all the negative PDMS replica layers were fabricated with thicknesses of approximately 0.4 mm. The photographs of the original plant leaf surfaces, which were taken with Nikon Eclipse Ci-S upright clinical microscopes, and their negative replicas are presented in Figure 2. The surface morphologies of the negative PDMS replica layers were similar in size and shape to the original leaf surfaces. The PDMS replication procedure resulted in accurate replication of the surfaces of the various leaves.

**Figure 3:**
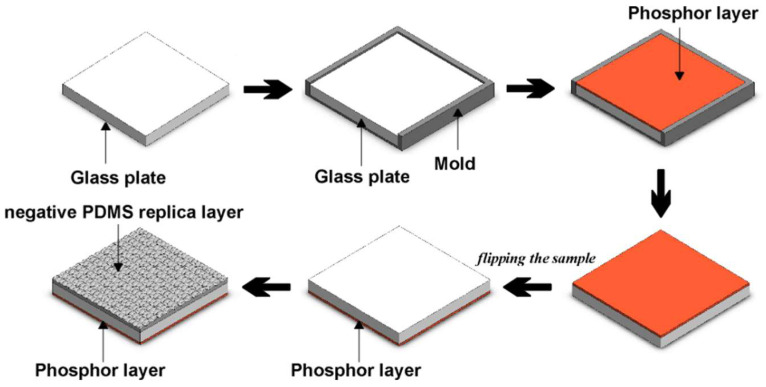
Schematic flowchart of fabrication of a semitransparent thin-film LSC with a negative replica layer of plant leaf microstructures.

To realize the application of the negative replica layers of plant leaf microstructures in semitransparent thin-film LSCs, the LSCs were fabricated using commercial phosphors [13]. Each LSC consisted of a glass plate (BA270) with dimensions of 50 mm × 50 mm × 5 mm, a phosphor layer, and a PV cell. The phosphor layer on the glass plate was fabricated by pouring the phosphor gel (a mixture of phosphor powder and silicone gel) into a rectangular aluminum mold on the glass plate. YAG4454-EL, RR6436, and LWR6931 inorganic phosphor powders from Intematix (Fremont, CA, USA) and silicone gel (OE-6370 HF) from Dow Corning were used to fabricate the thin phosphor layers of the LSCs. The thickness of the thin phosphor layer was set at 0.65 mm, and phosphor concentrations of 10%, 15%, 20%, 25%, and 30% were used. The glass plate with the phosphor layer was heated in an oven at 120 °C for 2 h and at 150 °C for 4 h to cure the phosphor layer on the glass plate. After the LSC was fabricated, the negative PDMS replica layer was attached to the top surface of the LSC with PDMS gel, as illustrated in Figure 3. The LSC with the negative PDMS replica layer was heated in an oven at 100 °C for 35 min to cure the PDMS gel between the negative PDMS replica layer and the glass plate. For comparison, a flat PDMS layer with the same thickness as the negative PDMS replica layer was also fabricated and attached to the top surface of a semitransparent thin-film LSC. Multicrystalline Si-PV cells with dimensions of 50 mm × 5 mm and an average power conversion efficiency of 14.7% were attached to the edges of the LSCs by using the PDMS gel. The cure time of optically clear silicone is 48 h at room temperature. In general, the use of opaque backside mirrors on the bottom surfaces of LSCs can increase the power conversion efficiency of LSCs. However, semitransparent and colorful LSCs are more easily incorporated into architectural designs than are their opaque counterparts. LSCs with backside mirrors are not appropriate for application in BIPV systems in which transparent and semitransparent windows are considered suitable candidates and were thus not fabricated in this study. Nevertheless, thin phosphor layers with high concentrations of phosphor are opaque. Accordingly, in this study, the phosphor concentrations of the thin phosphor layers were set at 10% to 30% to ensure the semitransparency of the LSCs.

**Figure 4:**
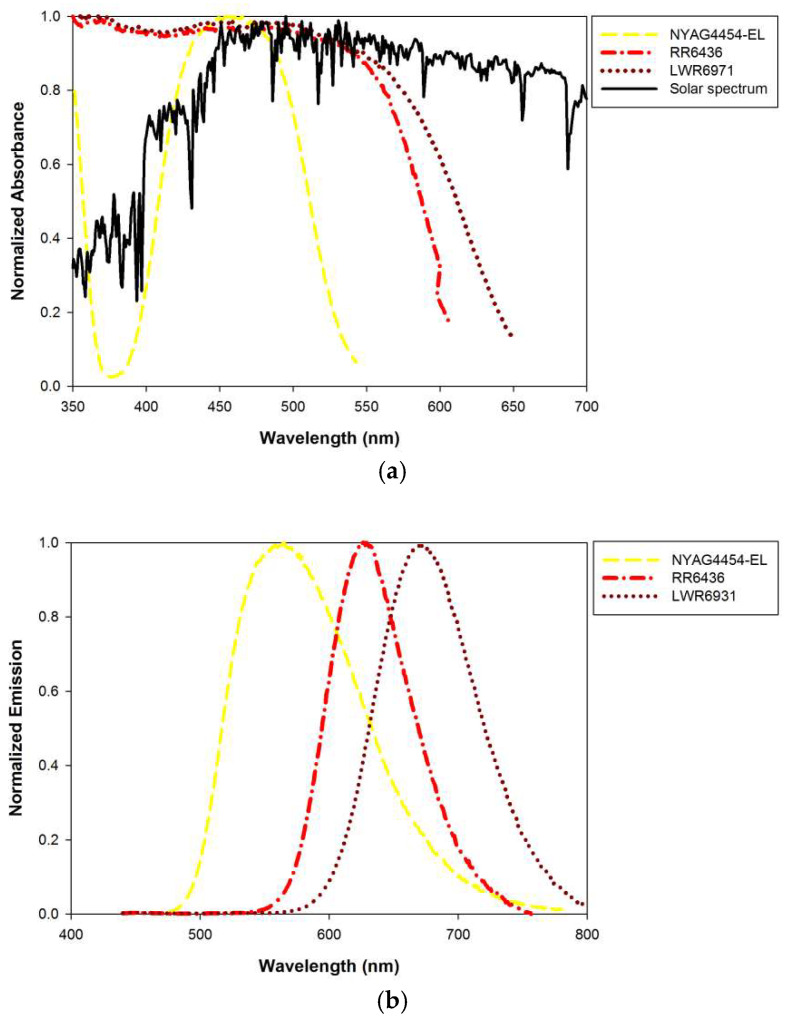
Normalized (**a**) excitation and (**b**) emission spectra of the phosphors used in this study.

The excitation spectra, emission spectra, and quantum yields of the YAG4454-EL, RR6436, and LWR6931 phosphor powders were measured using a FluoroMax 4 spectrometer (Horiba) with an integrating sphere. Figure 4a,b present the normalized excitation and emission spectra, respectively, of the phosphors. The semitransparent thin-film LSCs with different negative replica layers of leaf surface microstructures were tested using a solar simulator (Oriel 91160A, Newport, Irvine, CA, USA) under AM 1.5 (100 mW/cm^2^) conditions. A Keithley 2400 was used to determine the current–voltage curves of the PV cells attached to the LSCs with different negative replica layers of leaf surface microstructures.

## 3. Results and Discussion

**Figure 5:**
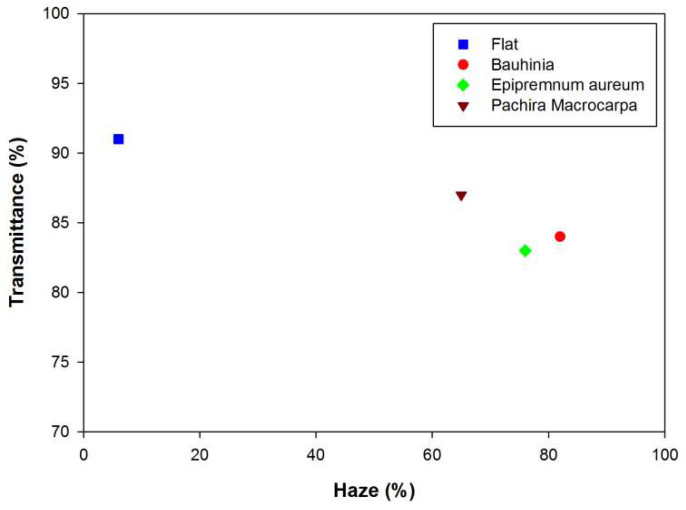
Transmittance versus haze ratio for the negative replica layers of *Bauhinia*, *Epipremnum aureum*, and *Pachira macrocarpa* leaf microstructures.

Multiple reflections on each air/encapsulation interface considerably dissipate the energy transmitted to a solar cell system, thereby decreasing the efficiency of a PV module. A glass shield or encapsulation layer with surface microstructures can reduce reflection on the air/encapsulation interface of a module, thereby enhancing its optical efficiency [14,15,16,17,18]. A glass shield or encapsulation layer with surface microstructures with high transparency and a high haze ratio is optimal. Incident light is scattered by the surface microstructures of the glass shield or encapsulation layer before it penetrates the PV module, resulting in enhanced light absorption by the PV cell. In this study, negative replica layers of leaf surface microstructures were used to increase the scattering of incident light on semitransparent thin-film LSCs containing commercial phosphors, thereby enhancing the optical efficiency of the LSCs. Optical haze, or the percent of light diffusely scattered through a transparent surface of the total light transmitted, is a useful parameter for assessing the effect of light scattering on the transparency of the negative replica layers of leaf surface microstructures. Higher haze can increase the path of light transmitted into the glass plate and thin phosphor layer of a LSC to improve the light absorption and emission of the phosphor, resulting in greater light collection by the PV cell on the edge of glass plate. Figure 5 illustrates the relationships between the optical transmittance and haze ratios of the negative replica layers of different leaf surface microstructures. The flat PDMS layer should be highly semitransparent with a low haze ratio; however, in this study, the surface microstructures on the negative replica layers increased the haze ratio and reduced transmittance. Among the negative replica layers with surface microstructures of different leaves, the layers with *Bauhinia* leaf microstructures had the highest haze ratios, followed by those with *Epipremnum aureum* and *Pachira macrocarpa* leaf microstructures. When light irradiates the negative replica layers with leaf surface microstructures, the transmitted light is scattered by the microstructures before it penetrates the layers of the LSC, causing the transmitted illumination spot to be larger than the incident beam. Furthermore, negative replica layers with higher haze ratios produce larger illumination spots.

The power conversion efficiency (PCE) is one of the key performances for PV device. The overall PCE of the LSC system can be defined as [23]:

(1) $$\eta_{\mathrm{LSC}}\;=\;\eta_{\mathrm{opt}}\cdot \eta_{\mathrm{PV}}=\frac{J_{\mathrm{SC}}\cdot V_{\mathrm{OC}}\cdot \mathrm{FF}}{P_{\mathrm{in}}}$$

where η_PV_ is the efficiency of the edge-mounted PV cell under the downshifted flux of the luminophore, η_opt_ is the optical collection efficiency, J_SC_ is the short-circuit current density, V_OC_ is the open-circuit voltage, FF is the fill factor, and P_in_ is the incident solar power density on the top surface of the LSC. Since the area receiving incident power is the front surface of the LSC, the measured short-circuit current density J_SC_ should be divided by the area of the LSC front surface. In this study, the relative optical collection efficiency η_opt_ is used to measure LSC performance and is defined as follows:

(2) $$\eta_{\mathrm{opt}}=\frac{\eta_{\mathrm{LSC}}}{\eta_{\mathrm{PV}}}$$

**Figure 6:**
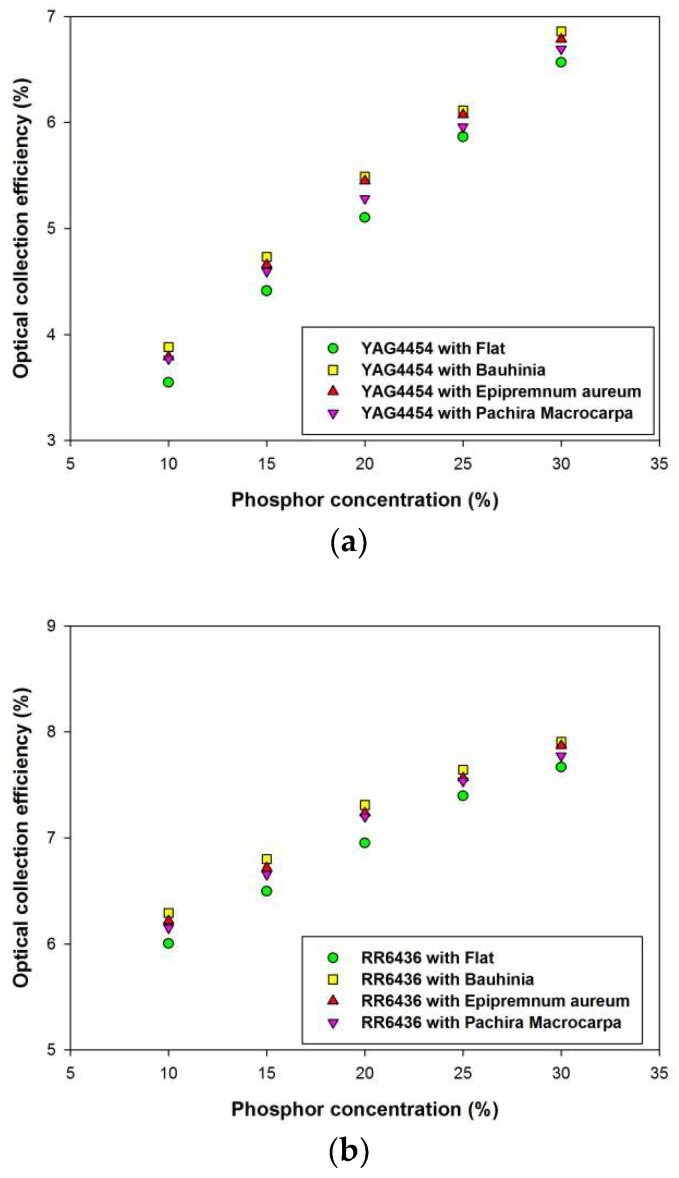

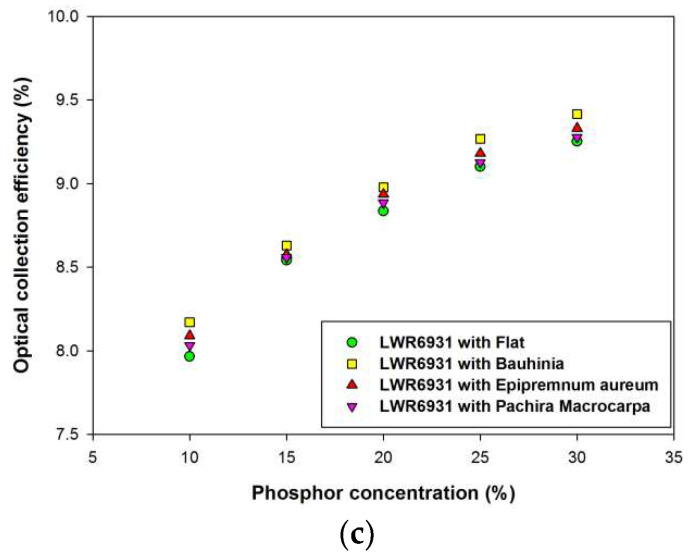
Optical collection efficiencies of semitransparent thin-film LSCs with (**a**) YAG4454, (**b**) RR6436, and (**c**) LWR6931 phosphor layers.

Figure 6a presents the optical collection efficiency η_opt_ of the LSCs with the negative replica layers of *Bauhinia*, *Epipremnum aureum*, and *Pachira macrocarpa* as a function of YAG4454-EL phosphor concentration. The optical collection efficiency η_opt_ increases as the phosphor concentration increases. The high fractions of light re-emitted by the thin phosphor layers with high concentrations increase the light trapped within the LSCs, thereby increasing the optical collection efficiencies of the LSCs. At any given phosphor concentration, the optical collection efficiency of the LSC with the negative replica layer of *Bauhinia* leaf microstructures was higher than those of the LSCs with other negative replica layers. The LSC with a negative replica layer of *Bauhinia* leaf microstructures exhibited the greatest optical efficiency, followed by those with negative replica layers of *Epipremnum aureum*, and *Pachira macrocarpa* leaf microstructures and the LSC with the flat PDMS layer, in that order. When the negative replica layer of a plant leaf is placed on the top surface of a glass plate, the negative replica layer facilitates the scattering of incident sunlight and increases the path of light transmitted into the glass plate and thin phosphor layer at the bottom surface of the LSC. Thus, the incident sunlight has a greater probability of interacting with the phosphor particles in the thin phosphor layer, thereby increasing the light available to the LSC PV cell, as illustrated in Figure 1. Similar to the results of the replication of leaf surface structures covered on PV cells, the negative replica layers with high haze ratios were able to scatter more incident sunlight, thereby increasing the optical collection efficiency of their respective LSCs [20]. Figure 5 presents the haze ratios of the negative replica layers of *Bauhinia*, *Epipremnum aureum*, and *Pachira macrocarpa*. The negative replica layer of *Bauhinia* leaf microstructures had the highest haze ratio, followed by those of *Epipremnum aureum*, and *Pachira macrocarpa* leaf microstructures and the flat PDMS layer, in that order.

Figure 6b,c illustrate the optical collection efficiencies η_opt_ of LSCs with negative replica layers on the top surface of the glass plate with different concentrations of RR6436 and LWR6931 phosphors, respectively. The characteristics of the semitransparent thin-film LSCs using inorganic phosphors RR6436 and LWR6931 were similar to those of the LSC using the YAG4454-EL phosphor. The optical collection efficiency η_opt_ of an LSC increases with phosphor concentration. At any given phosphor concentration, the optical collection efficiency of the LSC with the negative replica layer of *Bauhinia* leaf microstructures was higher than those of the LSCs with other negative replica layers. The LSC with a negative replica layer of *Bauhinia* leaf microstructures exhibited the greatest optical efficiency, followed by those with negative replica layers of *Epipremnum aureum*, and *Pachira macrocarpa* leaf microstructures and the LSC with the flat PDMS layer, in that order, which indicates that the use of negative replica layers of plant leaf can improve the optical collection efficiency of semitransparent thin-film LSCs regardless of the phosphor used in the thin phosphor films. However, the optical collection efficiency of the semitransparent thin-film LSC using LWR6931 was higher than those of the LSCs using RR6436 and YAG4454-EL because of the broad overlap of the LWR6931 absorption band and solar spectrum; greater overlap between the absorption band of the phosphor used in an LSC and the solar spectrum results in greater optical collection efficiency [13].

## 4. Conclusions

In this study, the effects of different negative replica layers of plant leaf microstructures used on semitransparent thin-film LSCs containing inorganic phosphor were investigated. Negative replica layers of plant leaf surface microstructures can reduce the high reflection on the air/glass interfaces of the glass plates in semitransparent thin-film LSCs, thereby enhancing the optical collection efficiency of the LSCs. Furthermore, a negative replica layer of plant leaf microstructures placed at the top surface of the glass plate can facilitate the scattering of incident sunlight and increase the path of light transmitted into the glass plate and thin phosphor layer at the bottom surface of an LSC. Thus, the incident sunlight has a greater probability of interacting with the phosphor particles in the thin-film phosphor layer, thereby increasing the amount of light collected by the PV cell on the edge of glass plate. At any given phosphor and phosphor concentration, the optical collection efficiency of the semitransparent thin-film LSCs with negative replica layers of *Bauhinia* leaf microstructures was higher than those of the LSCs with negative replica layers of *Epipremnum aureum*, and *Pachira macrocarpa* leaf microstructures. This difference is attributable to the higher haze ratio of the negative replica layers of *Bauhinia* leaf microstructures relative to those of the other negative replica layers; a higher haze ratio results in higher optical collection efficiency. However, the optical collection efficiency of the semitransparent thin-film LSC using LWR6931 was higher than those of the LSCs using RR6436 and YAG4454-EL because of the broad overlap of the LWR6931 absorption band and solar spectrum. The greater overlap between the absorption band of the LWR6931 and the solar spectrum results in greater optical collection efficiency. This study elucidates the effects of negative replica layers of plant leaf microstructures on the behavior of semitransparent thin-film LSCs containing inorganic phosphor, and the results of the study may inform guidelines for the design of new semitransparent thin-film LSCs.
